# Aconiti lateralis radix praeparata total alkaloids exert anti-RA effects by regulating NF-κB and JAK/STAT signaling pathways and promoting apoptosis

**DOI:** 10.3389/fphar.2022.980229

**Published:** 2022-09-02

**Authors:** Yeke Wu, Yi Liu, Lele Zhang, Lan Wen, Yunfei Xie

**Affiliations:** ^1^ Department of Stomatology, Hospital of Chengdu University of Traditional Chinese Medicine, Chengdu, Sichuan, China; ^2^ Department of Pharmacy, Clinical Medical College & Affiliated Hospital of Chengdu University, Chengdu, Sichuan, China; ^3^ School of Basic Medical Sciences, Chengdu University, Chengdu, Sichuan, China; ^4^ Department of Digestion and Endocrinology, Sichuan Provincial People’s Hospital Jinniu Hospital, Chengdu, Sichuan, China; ^5^ Department of Nuclear Medicine, Sichuan Provincial People’s Hospital, University of Electronic Science and Technology of China, Chengdu, Sichuan, China

**Keywords:** aconiti lateralis radix praeparata, anti-inflammation, apoptosis, cell cycle, total alkaloids

## Abstract

Aconiti Lateralis Radix Praeparata (“Fuzi” in Chinese) is one of the traditional herbs widely used to intervene rheumatoid arthritis (RA), while Fuzi total alkaloids (FTAs) are the main bioactive components. However, the treatment targets and specific mechanisms of FTAs against RA have not been fully elucidated. The purpose of the present study was to confirm the anti-rheumatism effects of FTAs and reveal its potential molecular mechanisms. In TNF-*α*-induced MH7A cells model, we found that FTAs showed inhibitory effects on proliferation. While, FTAs significantly decreased the expression levels of IL-1*β*, IL-6, MMP-1, MMP-3, PGE2, TGF-*β,* and VEGF. FTAs also enhanced the progress of apoptosis and arrested the cell cycle at G0/G1 phase to prevent excessive cell proliferation. In addition, FTAs inhibited the hyperactivity of NF-κB and JAK/STAT signaling pathways, and regulated the cascade reaction of mitochondrial apoptosis signaling pathway. The results suggested that FTAs exerted anti-inflammatory effects by inhibiting NF-κB and JAK/STAT signaling pathways, promoted apoptosis by stimulating mitochondrial apoptosis signaling pathway, and inhibited cell proliferation by modulating cell cycle progression.

## Introduction

Rheumatoid arthritis (RA) is an autoimmune disease characterized by a systemic immune inflammatory response, which can accompany synovial tissue hyperplasia, joint swelling, bone destruction, chronic pain, and even loss of functions. The patient’s living situation is severely deteriorated with the progress of RA (Philippou et al., 2021). The etiology of RA is not very clear, but studies have suggested that it may be related to familial inheritance, estrogen level, environmental factors, social stress, pathogen infection, gut microbiota metabolites and other factors (Liu and Xie, 2021; Liu et al., 2022). Statistics show that about 0.5%–1.0% of the worldwide population is diagnosed with RA, while according to the data of the Chinese Medical Association, the prevalence rate of RA in China is nearly 0.42%, and the prone age incidence is 30–50 years old, with the number of female patients almost 4 times than that of male patients (Huang et al., 2021). In clinical treatment, disease-modifying antirheumatic drugs (DMARDs), biological targeting agents, nonsteroidal anti-inflammatory drugs (NSAIDs), glucocorticoids are the most frequently applied anti-RA drugs (Burmester and Pope, 2017). In recent years, Chinese herbal extracts including “Total Glucosides of White Paeony Capsules” and “Tripterygium Glycoside Tablets” have been extensively utilized and proved to achieve better effectiveness (Zhang and Wei, 2020; Zhang Y et al., 2021). In general, traditional Chinese medicine (TCM) shows a potent and lasting role in the treatment of RA.

As the processed and dried lateral root of *Aconitum carmichaelii* Debx. Aconiti Lateralis Radix Praeparata (“Fuzi” in Chinese) was first recorded in “Sheng Nong’s herbal classic,” and has a long history of clinical application in China. In the theoretical system of TCM, Fuzi is often combined with other herbs to form a common multi-drug combination pattern, which appears in numerous TCM medical preparations, such as “Shaoyao-Gancao-Fuzi Decoction,” “Guizhi-Shaoyao-Zhimu Decoction,” and recommended for the treatment of RA (Zhang Q et al., 2021). Accumulating research evidences have proven that the water extract of Fuzi could effectively resist rheumatism, reduce swelling and relieve pain (Yang et al., 2018). The chemical constituents of Fuzi mainly include alkaloids, polysaccharides and organic acids. Among them, C19-diterpenoid alkaloids, C20-diterpenoid alkaloids and non-diterpenoid alkaloids are the major bioactive components of Fuzi (Zhao et al., 2020). However, some C19-diterpenoid alkaloids with diester structure, such as aconitine, hypaconitine and mesaconitine, have a small safe dose range (Liu et al., 2020). Previous studies have shown that the alkaloids extract from Fuzi exhibit strong anti-RA activity in animal and cell experiments, such as inhibiting the expression of pro-inflammatory cytokines, alleviating arthritis symptoms of adjuvant-induced arthritis (AIA) model rats, and participating in the regulation of some inflammatory related signaling pathways (Xie et al., 2019; Zhang L et al., 2021; Guo et al., 2022). Therefore, the alkaloids extract such as Fuzi total alkaloids (FTAs) is suggest to be the main effective components in the treatment of RA.

Through previous *in vivo* experiments, we found that the alkaloids extract from Fuzi could reduce paws swelling and arthritis scores, inhibit serum levels of IFN-*γ*, TNF-*α,* and IL-1*β*, reverse the imbalance of energy and amino acids metabolism, and showed strong anti-inflammatory activity and even toxic side effects in AIA rats (Xie et al., 2019). Moreover, according to the above metabolomics results, it was speculated that the alkaloids extract from Fuzi might regulate metabolic disorder of linoleate by inhibiting the activation of NF-κB signaling pathway. However, the treatment targets and specific mechanisms need to be further explored. Consequently, the present study was committed to verifying the anti-rheumatism effects of FTAs in TNF-*α*-induced MH7A cells and illuminating the pharmacological mechanisms involved, as well as explaining the scientific connotation of Fuzi in the treatment of RA based on the TCM theory.

## Materials and methods

### Materials

MH7A cells were obtained from Jennio Biotech Co., Ltd. (Guangzhou, China). TNF-*α* was obtained from Novoprotein Biotechnology Co., Ltd. (Shanghai, China). DMEM/high glucose, PBS, PMSF, RIPA lysis buffer, phosphatase and protease inhibitor, SDS-PAGE gel kit, goat anti-rabbit IgG-HRP, histone H3, TBS buffer, Cell membrane-breaking kit, DAPI, antifade mounting medium, JAK2, Bax, IκB*α* were obtained from Servicebio Co., Ltd. (Wuhan, China). Trypsin 0.25% (1X) solution, penicillin-streptomycin were obtained from HyClone Co., Ltd. (Logan, Utah, United States). FBS was obtained from Merck/Sigma Co., Ltd. (St. Louis, Missouri, United States). Commercially available ELISA kits for IL-1*β*, IL-6, MMP-1, MMP-3, PGE2, VEGF, TGF-*β,* and Annexin V-FITC and PI dual-staining apoptosis kit were obtained from Elabscience Biotechnology Co., Ltd. (Wuhan, China). BCA protein quantitative kit, protein loading buffer, ECL chemiluminescence kit (enhanced), CCK-8 kit were obtained from Biosharp Co., Ltd. (Hefei, China). Tween 20, BSA, glycine were obtained from Bioforxx Co., Ltd. (Germany). Bcl-2, caspase-3, p-p65, *β*-actin were obtained from Affinity bioscience Co., Ltd. (Cincinnati, Ohio, United States). IKK*β*, p-IKK*β* were obtained from Abcam Co., Ltd. (Cambridge, United Kingdom). p-IκB*α*, STAT3 were obtained from Biosynthesis Biotechnology Inc. (Beijing, China). P65 was obtained from Proteintech Group, Inc. (Chicago, Illinois, United States). Cell cycle staining kit and cell membrane-breaking kit were obtained from Multisciences (Lianke) Biotech Co., Ltd. (Hangzhou, China). Cy3-labeled goat anti-rabbit IgG was obtained from Beyotime Biotechnology Co., Ltd. (Shanghai, China). TPCA-1 was obtained from Selleck Chemicals Co., Ltd. (Houston, Texas, United States).

### Extraction and identification of Fuzi total alkaloids

The raw Fuzi from Good Agricultural Practice (GAP) planting base was provided by Zhongba Fuzi Co., Ltd. (Jiangyou, China). 3 kg of raw Fuzi was soaked in 0.1 mol/L hydrochloric acid (1:8, v/v) for 3 h and heated for extraction twice, 2 h each time. Then, we concentrated the extract to about 1 g/ml, adjusted the PH to 3, and passed it through styrene strong acid cation exchange resin at a flow rate of 5–6 BV/h. After adsorption, the extract was washed with distilled water to remove impurities. Immediately, a mixture of 70% ethanol containing 9% ammonia was eluted at a flow rate of 2-3 BV/h until there was no color reaction between the effluent and bismuth potassium iodide. Finally, the eluent was collected and concentrated to obtain about 7.0 g of FTAs.

The quantitative analysis of FTAs was accomplished in UPLC-MS system with Waters ACQUITY BEH C18 column (2.1 × 50 mm, 1.7 μm). The mobile phase of chromatographic separation was consisted of solvent A (acetonitrile) and solvent B (water, containing 0.1% formic acid). The gradient elution conditions was set as follows: 80%–0% B at 0–5 min, 0% B at 5–7 min, 0%–80% B at 7–8 min, 80% B at 8–10 min. The flow rate was set at 0.3 ml/min and the column temperature was maintained at 35°C. After equilibration, 0.2 μl sample was injected for detection.

**FIGURE 1:**
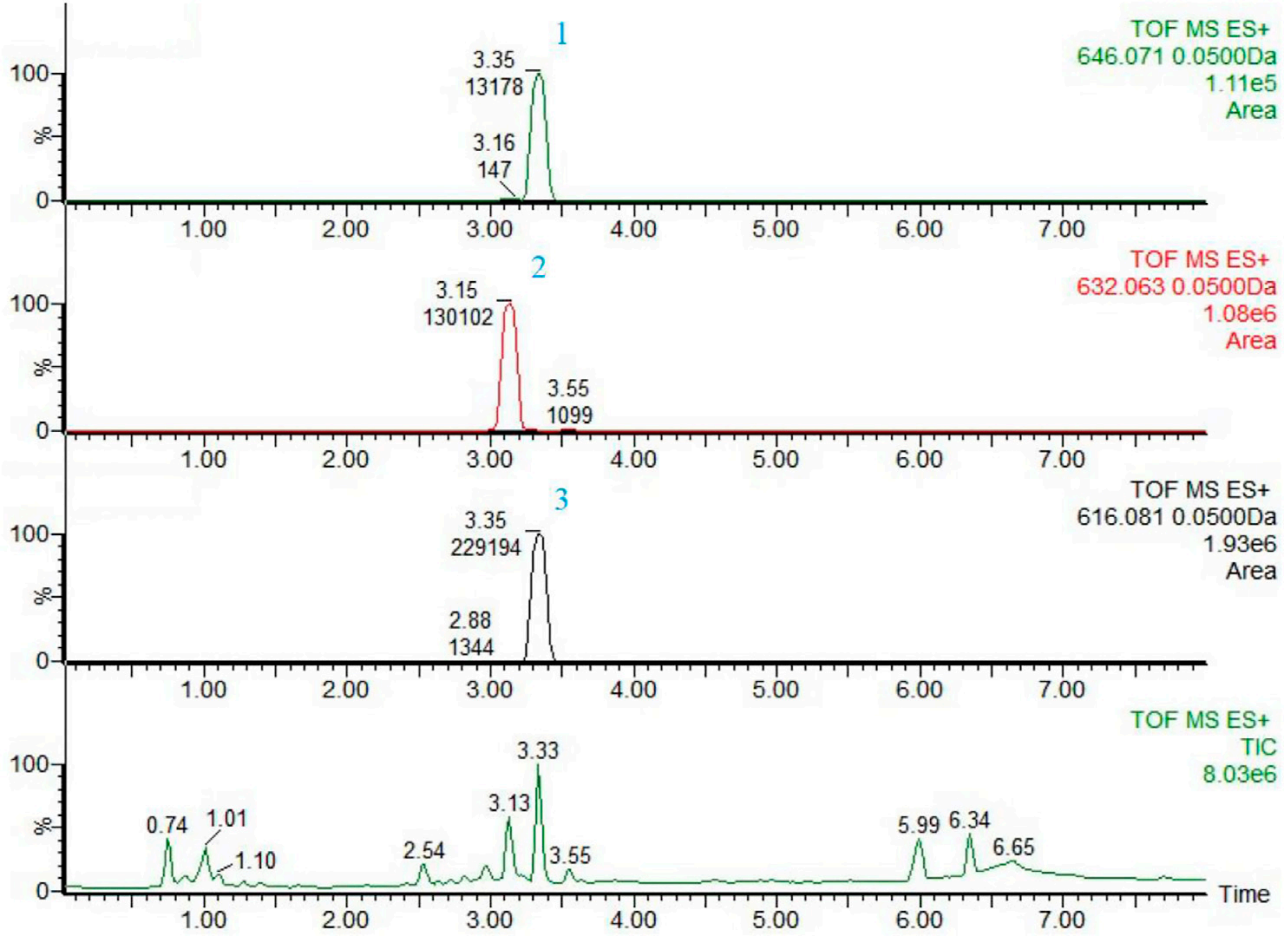
The chemical fingerprints and analysis results of FTAs by UPLC-MS. 1) Aconitine, 2) Mesaconitine, and 3) Hypaconitine.

Aconitine, hypaconitine and mesaconitine were selected as the reference compounds for contents determination of FTAs, and they were 4.71 μg/mg, 59.11 μg/mg and 35.22 μg/mg in concentration, respectively. The chemical fingerprints of FTAs were displayed in Figure 1.

### Cell culture

MH7A cells are highly consistent with the pathologic characteristics of parental fibroblast-like synoviocytes (FLSs), and they are a kind of mature immortalized FLS line widely used in RA studies. MH7A cells were sub-cultured in DMEM/high glucose containing 15% FBS and 1% penicillin-streptomycin. The environment was kept constant at 5% CO_2_ and 37°C, with saturated humidity. MH7A cells were passaged approximately every 3 days, and the 3-9 generations were used for all experiments. FTAs were dissolved in 0.1% DMSO and prepared to the corresponding concentrations with culture medium. The Ethics Committee of Sichuan Provincial People’s Hospital reviewed the experimental plan of this study.

### Cell viability assay

Due to the high toxicity of FTAs, it is necessary to determine the effects of FTAs on MH7A cells viability via CCK-8 assay. MH7A cells were incubated in 96-well plate with the density of approximately 5×10^3^/well for 24 h, followed by intervention with the different concentrations of FTAs (1, 10, 50, 100, 200 μg/ml). After this, 10 μl CCK-8 reagent was supplemented into all the culture wells and the cells were incubated for another 3 h, at 37°C. Finally, at 450 nm wavelength, the optical density (OD) value of the microtiter plate was detected by the microplate reader. (drug_[OD]_-blank_[OD]_)/(control_[OD]_-blank_[OD]_) × 100% was used to calculate the viability of MH7A cells.

### ELISA examinations

MH7A cells were incubated in 96-well plate, then exposed to 20 ng/ml TNF-*α* and treated with low, medium and high concentrations of FTAs (1, 10, 100 μg/ml) for 24 h. After incubation, the supernatant samples of MH7A cells were collected and the contents of IL-1*β*, IL-6, MMP-1, MMP-3, PGE2, TGF-*β*, and VEGF were detected by ELISA kits. In addition, MH7A cells were incubated with 0.5 μM TPCA-1 for 24 h, and then cultured with 20 ng/ml TNF-*α* and 1 μg/ml FTAs for another 24 h. Finally, the contents of IL-1*β* and IL-6 were detected by ELISA kits.

### Western blot analysis

The total proteins of MH7A cells were obtained after treatment with RIPA lysis buffer supplemented with a mixture of phosphatase and protease inhibitors. After centrifugation, the supernatants were collected and transferred to BCA protein quantitative kit for protein concentration detection. Before transferring to PVDF membranes, the equal amount of protein samples were fractionated by 10% SDS-PAGE. Subsequently, the TBS-T buffer containing 5% skimmed milk was selected to block the PVDF membranes for 1.5 h. Immediately, the membranes were coated with the diluted primary antibodies of IκB*α* (rabbit, diluted to 1/1,000), p-IκB*α* (rabbit, diluted to 1/1,000), IKK*β* (rabbit, diluted to 1/2,000), p-IKK*β* (rabbit, diluted to 1/1,000), p65 (rabbit, diluted to 1/1,000), p-p65 (rabbit, diluted to 1/1,000), Bcl-2 (mouse, diluted to 1/1,000), Bax (rabbit, diluted to 1/1,000), caspase-3 (rabbit, diluted to 1/1,000), JAK2 (rabbit, diluted to 1/1,000), STAT3 (rabbit, diluted to 1/1,000) and *β*-actin (rabbit, diluted to 1/5,000), and incubated overnight at 4°C. Following this, the incubation was continued with HPR-conjugated antibody (goat anti-rabbit IgG-HRP, diluted to 1/10,000) at room temperature for another 1 h. Lastly, the protein signals were displayed after being processed by ECL kit, and the gray levels of the bands were read and recorded with ImageJ software.

### Immunofluorescence assay

For immunofluorescence assay, MH7A cells were placed on the slides, treated with BPS-Triton X-100 (0.1%) mixed reagent for 20 min, and blocked with serum-supplemented medium at room temperature for 35 min. Immediately, the primary antibodies were diluted to the corresponding concentrations with PBS, and the cells were treated with p-p65, Bax overnight at 4°C. Then, the Cy3-labeled goat anti-rabbit IgG was added and treated for another 50 min. Subsequently, the cells were washed twice with PBS and further incubated with DAPI fluorescent dye for 10 min. Finally, the Nikon fluorescence microscope system was selected to photograph the above slides, and the fluorescence intensity was recorded with Image-Pro Plus v.6.0 (Media Cybernetics, Inc. Silver spring, MD, United States) processing program.

### Flow cytometry analysis of apoptosis and cell cycle

Before the experiments, the MH7A cells were washed and centrifuged to discard the supernatants. For apoptosis assay, 500 μl diluted 1×Annexin V binding buffer was selected to re-suspend the MH7A cells. Immediately, 3 μl of Annexin-V-FITC and PI staining reagents were supplemented to the cell suspensions, respectively. After being mixed, the cells were incubated at room temperature for 15 min, under light-avoiding condition. Then, the apoptosis rate of MH7A cells was detected by Bechman Coulter flow cytometer. For cell cycle assay, the MH7A cells were treated with 1 ml DNA staining reagent and 10 μl permeabilization reagent. After being mixed, the cells were incubated at room temperature for 30 min, under light-avoiding condition. Then, the cell cycle of MH7A cells was detected by Bechman Coulter flow cytometer. The numbers of gap 1- (G1-), synthesis- (S-), and gap 2/mitotic- (G2/M-) phase cells were quantified using Modfit cell cycle analysis software.

### Statistical analysis

All the experimental results were the average of three repeated experiments, and recorded as mean ± SD. SPSS 25.0 (IBM, Chicago, Illinois, United States) software was applied to perform statistical analysis of the raw data. The difference analysis among the multiple groups was carried out by One-Way ANOVA and Student’s *t*-test, and *p* < 0.05 was regarded to represent statistically significant.

## Results

### Effects of Fuzi total alkaloids on MH7A cells viability

**FIGURE 2:**
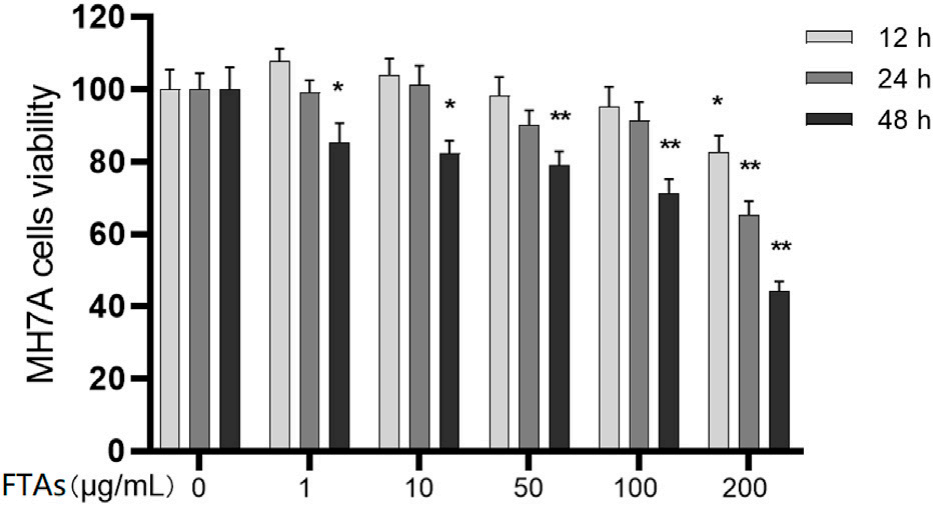
The effects of FTAs on MH7A cells viability. MH7A cells were incubated in microtiter plate and intervened by different concentrations of FTAs for 12, 24, and 48 h, and then the cells viability was assessed by CCK-8 method. The experimental results were recorded as mean ± SD (*n* = 3). *p < 0.05, **p < 0.01 vs. normal.

Due to the high toxicity of FTAs, medication safety must be ensured first. The MH7A cells were intervened by different concentrations of FTAs for 12, 24, and 48 h, and the cytotoxicity of FTAs was detected by CCK-8 method. As demonstrated in Figure 2, the cells viability was not significantly inhibited with the concentrations of FTAs no higher than 100 μg/ml at 12 and 24 h, except for 48 h. However, significant cytotoxicity was exhibited with 200 μg/ml FTAs at 12 h. Based on the above results, FTAs with concentrations of 1, 10, and 100 μg/ml were selected for the subsequent experiments, and the treatment duration was no more than 24 h.

### Fuzi total alkaloids inhibited IL-1*β*, IL-6, MMP-1, MMP-3, PGE2, TGF-*β* and VEGF secretion in TNF-*α*-induced MH7A cells

**FIGURE 3:**
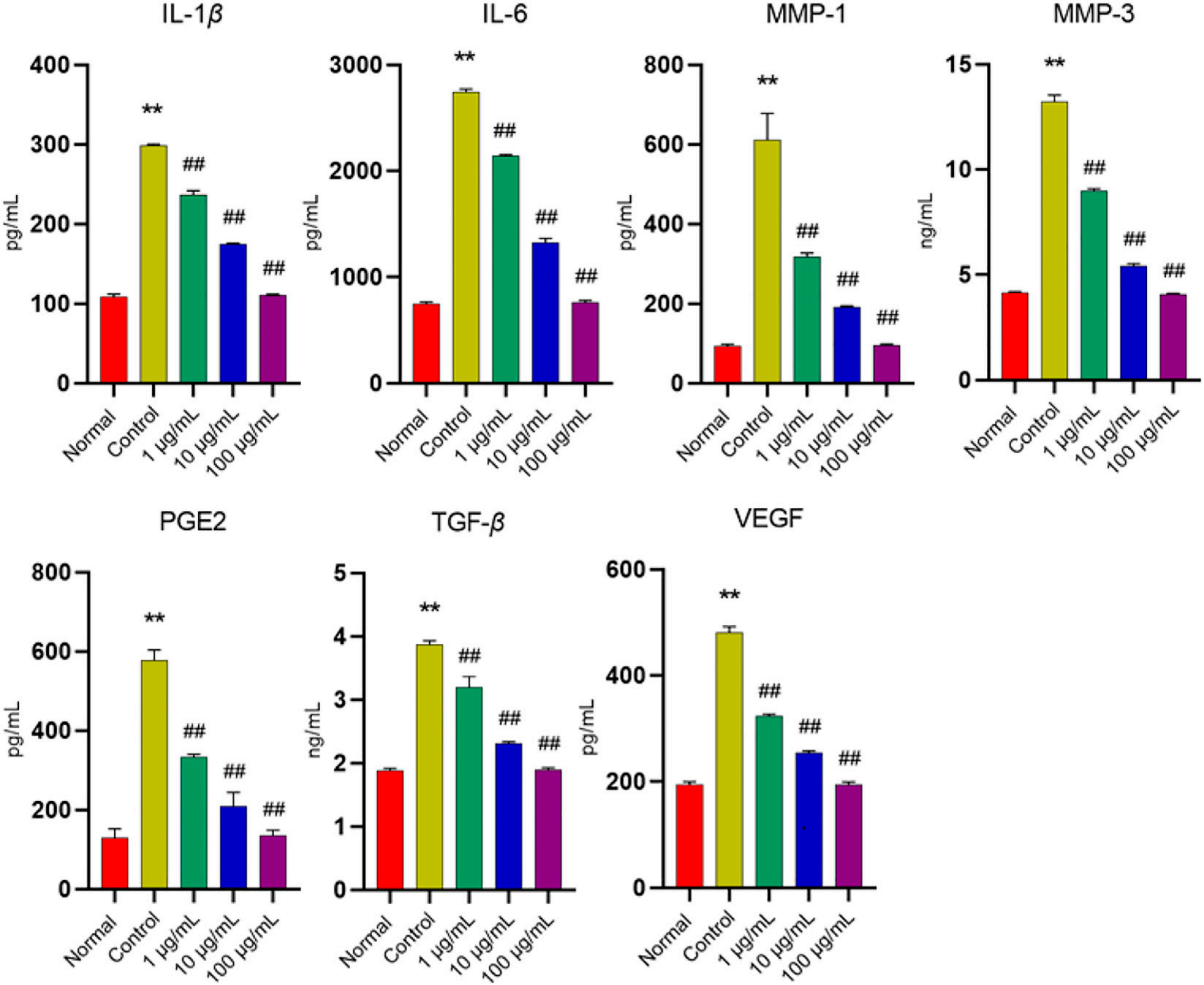
The effects of FTAs on the contents of IL-1*β*, IL-6, MMP-1, MMP-3, PGE2, TGF-*β* and VEGF in TNF-*α*-induced MH7A cells. MH7A cells were intervened by corresponding concentrations of FTAs in absence or presence of 20 ng/ml TNF-*α* for 24 h, and then the levels of the above inflammatory mediators were detected by the corresponding ELISA kits. The experimental results were recorded as mean ± SD (*n* = 3). *p < 0.05, **p < 0.01 vs. normal, ^#^ *p* < 0.05, ^##^ *p* < 0.01 vs. control.

The imbalance of cytokines expression is closely associated with the autoimmune inflammation of RA. Therefore, some biological agents play an anti-RA role by targeting the relevant cytokines. However, whether FTAs can regulate the expression of cytokines needs to be further explored, and in the present study, the effects of FTAs on TNF-*α*-induced inflammatory mediators were analyzed by ELISA assay. As demonstrated in Figure 3, compared with untreated MH7A cells, TNF-*α* significantly boosted the levels of IL-1*β*, IL-6, MMP-1, MMP-3, PGE2, TGF-*β,* and VEGF. While the various concentrations of FTAs could significantly reverse the TNF-*α-*induced augmented expression of the above inflammatory mediators. In conclusion, the results suggested that FTAs could significantly inhibit inflammatory responses in MH7A cells induced by TNF-*α*.

### Fuzi total alkaloids suppressed hyperactivity of NF-κB and JAK/STAT signaling pathways in TNF-*α*-induced MH7A cells

**FIGURE 4:**
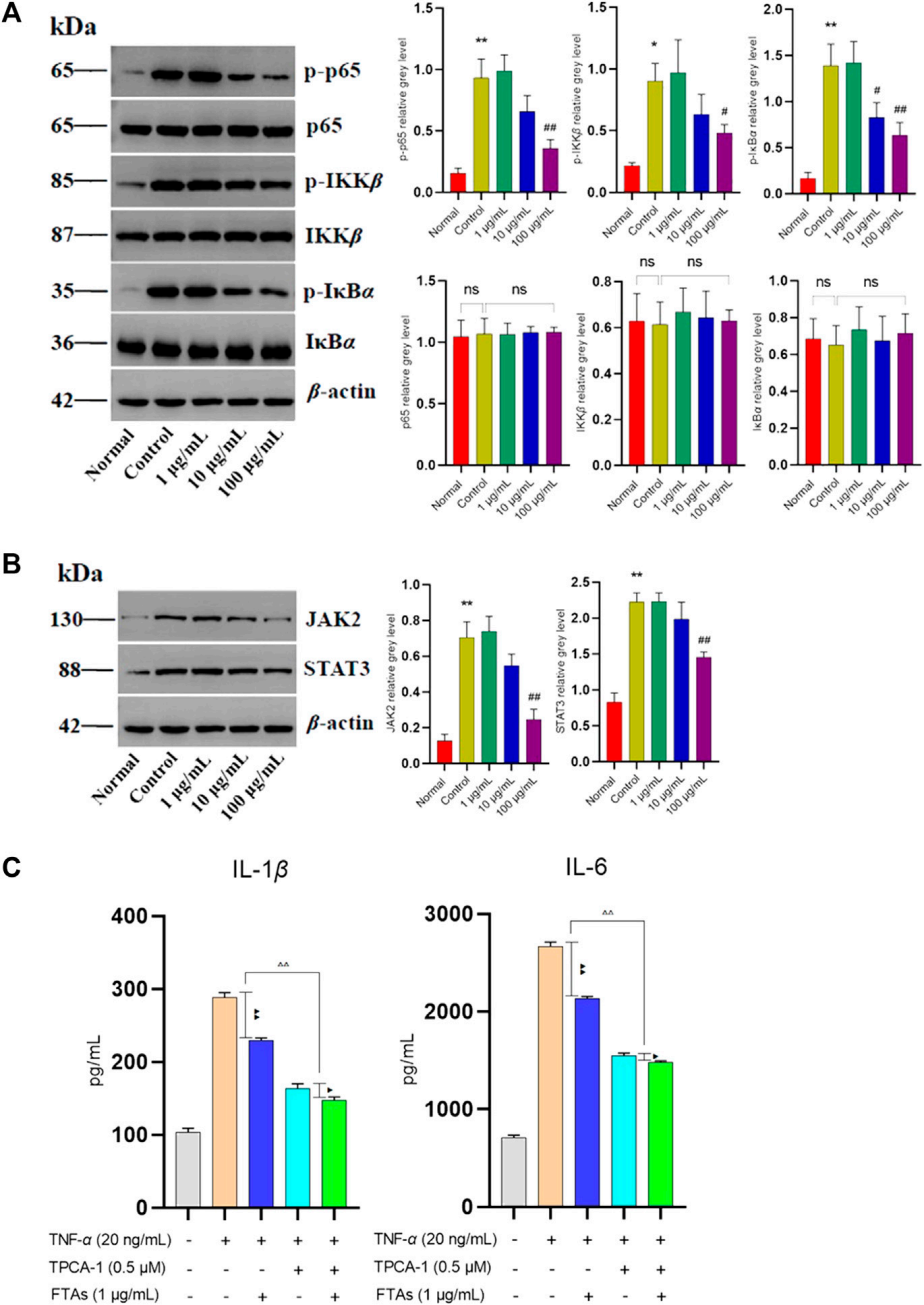
The effects of FTAs on activation of NF-κB and JAK/STAT signaling pathways in TNF-*α*-induced MH7A cells. MH7A cells were intervened by corresponding concentrations of FTAs in absence or presence of 20 ng/ml TNF-*α* for 24 h, and then the protein signal intensity of p-p65, p65, p-IKK*β*, IKK*β*, p-IκB*α*, and IκB*α* in NF-κB **(A)** and JAK2, STAT3 in JAK/STAT **(B)** signaling pathways were measured by western blot. **(C)** The inhibitory effects of FTAs on IL-1*β* and IL-6 in absence or presence of TPCA-1 were measured by ELISA kits. The experimental results were recorded as mean ± SD (*n* = 3). *p < 0.05, **p < 0.01 vs. normal, ^#^ *p* < 0.05, ^##^ *p* < 0.01 vs. control, ^△^ *p* < 0.05, ^△△^ *p* < 0.01, ^▲^ *p* < 0.05, and ^▲▲^ *p* < 0.01.

Extensive clinical evidences have shown that the hyperactivity of NF-κB and JAK/STAT signaling pathways may lead to inflammation, and is closely related to the pathophysiological processes of RA. However, whether FTAs play an anti-RA role by regulating the above signaling pathways needs to be further confirmed. Therefore, the effects of FTAs on protein expressions of NF-κB and JAK/STAT signaling pathways were assessed using western blot. As expected, for NF-κB signaling pathway analysis (Figure 4A), TNF-*α* stimulation significantly upregulated the contents of phosphorylated p65, IKK*β* and IκB*α*, while FTAs could abolish the abnormal phosphorylation of p65 (except for 1, 10 μg/ml), IKK*β* (except for 1, 10 μg/ml) and IκB*α* (except for 1 μg/ml). Furthermore, TNF-*α* stimulation also significantly increased the protein signal intensity of JAK2 and STAT3, while high concentration of FTAs significantly decreased the protein signal intensity of JAK2 and STAT3 (Figure 4B). To reveal the relevant evidence of FTAs in regulating NF-κB and JAK/STAT signaling pathways, TPCA-1, a dual specific inhibitor of IKKs and STAT3, was employed to block the TNF-*α*-induced activation of NF-κB and JAK/STAT signaling pathways. After further treatment with FTAs, IL-1*β* and IL-6 were selected to confirm whether the anti-inflammatory mechanism of FTAs was directly related to regulating the above two signaling pathways. The statistical results showed that when the activities of IKKs and STAT3 were blocked by TPCA-1, the inhibitory effects of FTAs on IL-1*β* and IL-6 were significantly weakened compared with the TNF-*α*-FTAs group without inhibitor. In addition, when TPCA-1 was considered as a positive control group, it also showed that FTAs and TPCA-1 had the same regulatory effects on IL-1*β* and IL-6 (Figure 4C). These results indicated that FTAs had anti-inflammatory effects, while NF-κB and JAK/STAT were the main regulatory pathways.

**FIGURE 5:**
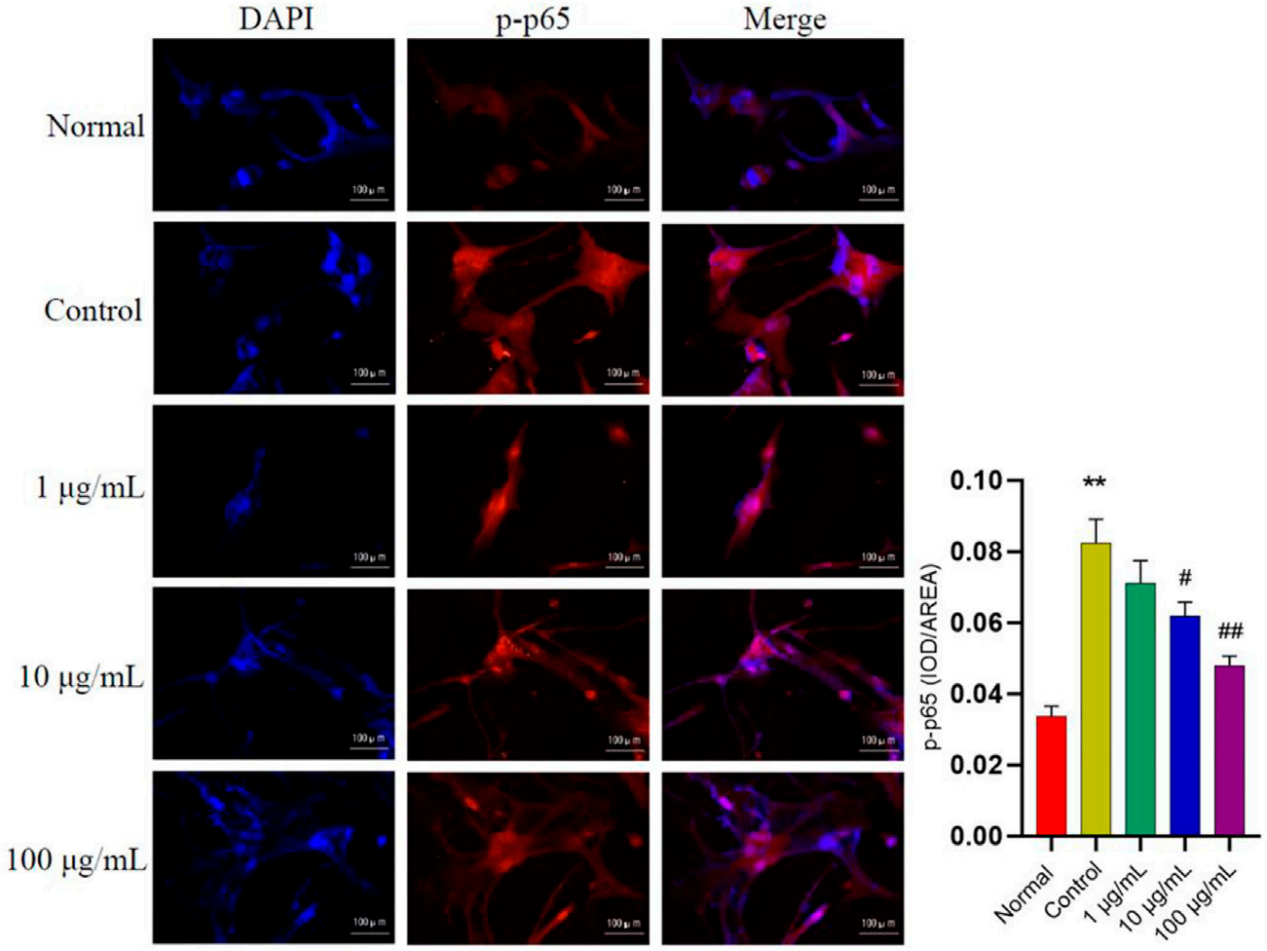
The effects of FTAs on expression of p-p65 in the nucleus of TNF-*α*-induced MH7A cells. MH7A cells were intervened by corresponding concentrations of FTAs in absence or presence of 20 ng/ml TNF-*α* for 24 h, and then the immunofluorescence of p-p65 in the nucleus were photographed by Nikon fluorescence microscope system. The experimental results were recorded as mean ± SD (*n* = 3). *p < 0.05, **p < 0.01 vs normal, ^#^ *p* < 0.05, ^##^ *p* < 0.01 vs. control.

In addition, to further verify the effects of FTAs on NF-κB signaling pathway, immunofluorescence staining was applied to analyze the level of p-p65 within the nucleus of MH7A cells. As demonstrated in Figure 5, medium and high concentrations of FTAs significantly decreased the expression of p-p65 in the nucleus, indicating that TNF-*α* stimulated NF-κB nuclear translocation was significantly suppressed.

### Fuzi total alkaloids promoted apoptosis by regulating related proteins of mitochondrial apoptosis signaling pathway

**FIGURE 6:**
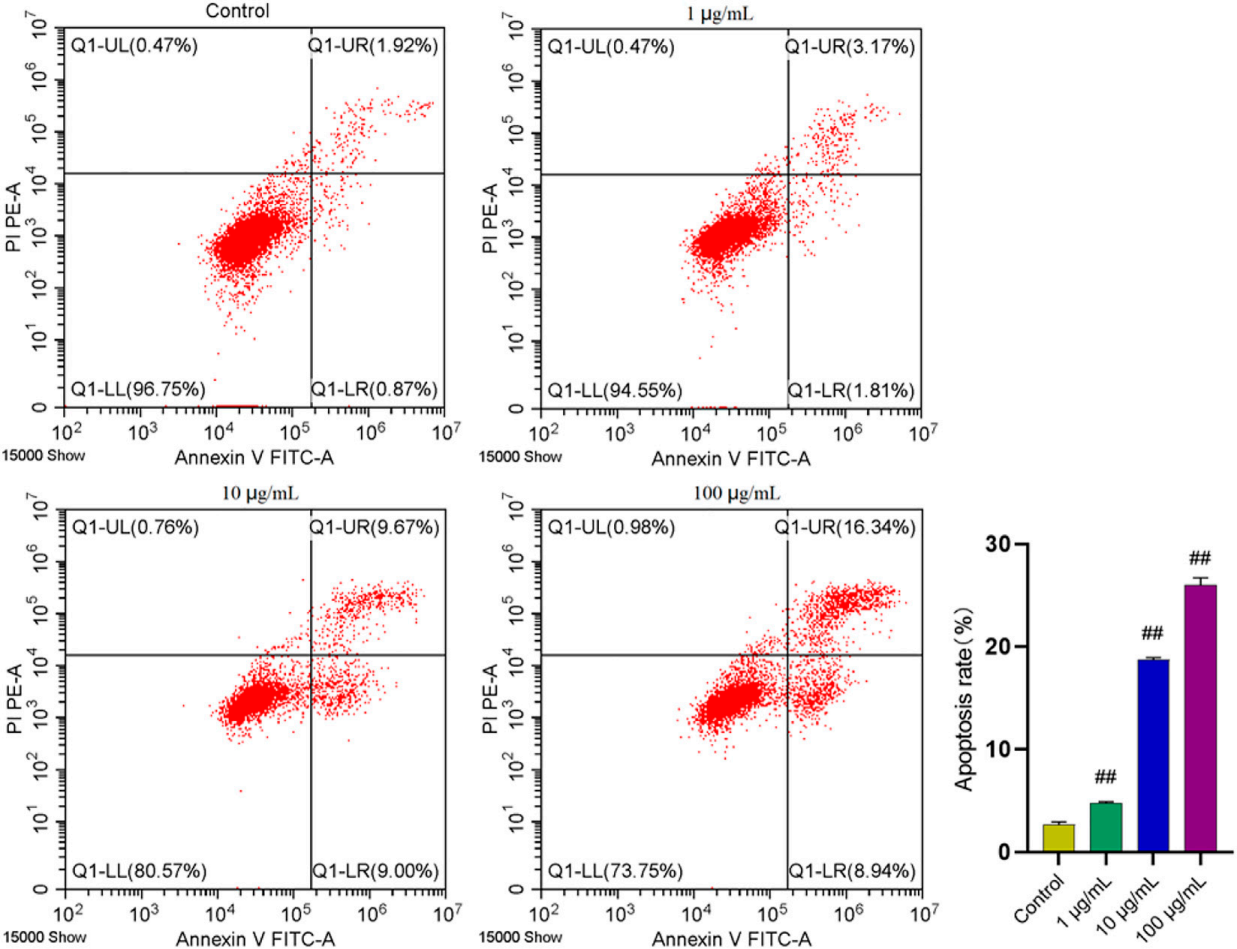
The effects of FTAs on apoptosis of TNF-*α*-induced MH7A cells. MH7A cells were exposed to 20 ng/ml TNF-*α* and intervened by corresponding concentrations of FTAs for 24 h, and then the apoptosis of MH7A cells was measured by Annexin V-FITC and PI double staining and flow cytometer. The experimental results were recorded as mean ± SD (*n* = 3). ^#^ *p* < 0.05, ^##^ *p* < 0.01 vs. control.

Inducing apoptosis of the excessive synovial cells is another major approach for RA treatment. Hence, flow cytometry was used to analyze whether FTAs could induce apoptosis of MH7A cells, thus exerting anti-RA effects. As demonstrated in Figure 6, FTAs could significantly promote apoptosis of TNF-*α*-induced MH7A cells manifested with Annexin V+/PI- and V+/PI+, no matter the cells were in early or late stage of apoptosis.

**FIGURE 7:**
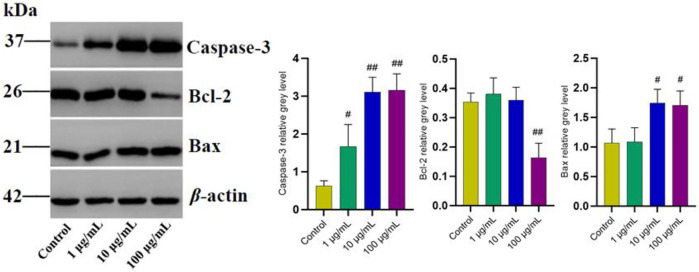
The effects of FTAs on mitochondrial apoptosis signaling pathway in TNF-*α*-induced MH7A cells. MH7A cells were exposed to 20 ng/ml TNF-*α* and intervened by corresponding concentrations of FTAs for 24 h, and then the protein signal intensity of caspase-3, Bcl-2 and Bax were measured by western blot. The experimental results were recorded as mean ± SD (*n* = 3). ^#^ *p* < 0.05, ^##^ *p* < 0.01 vs. control.

**FIGURE 8:**
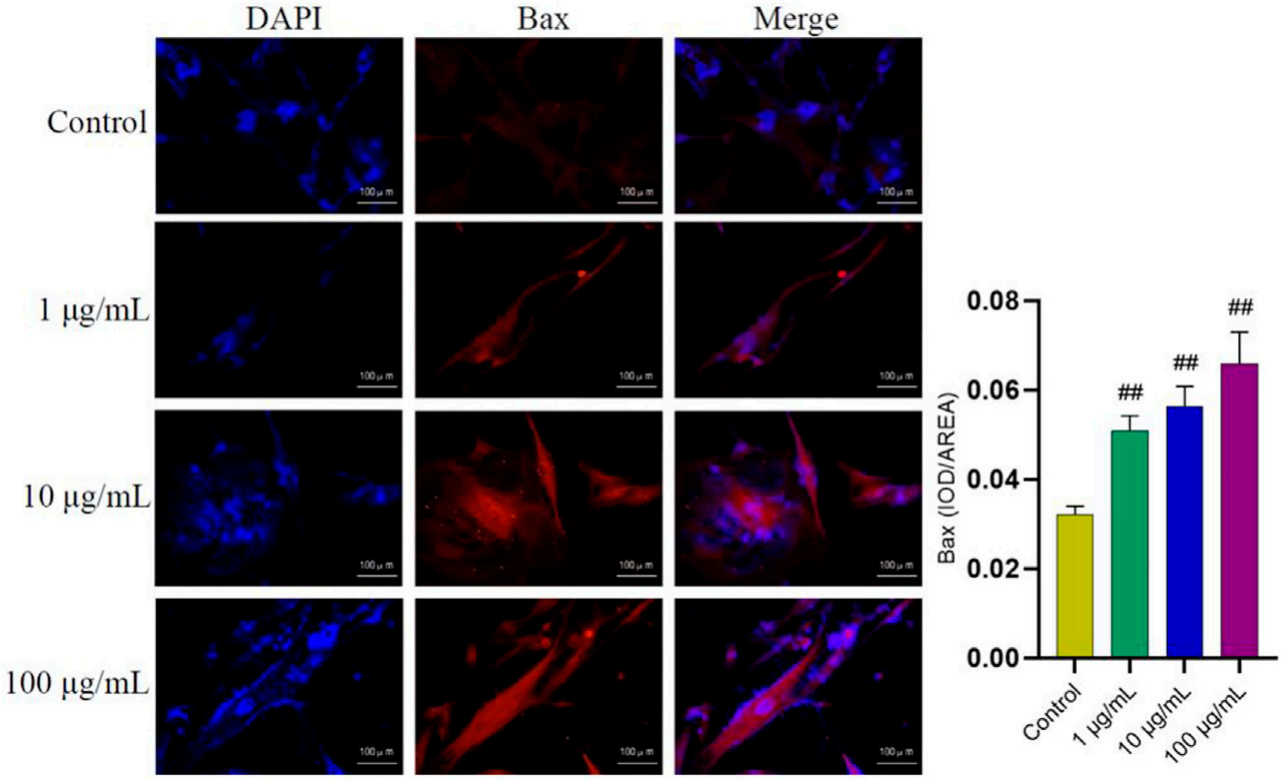
The effects of FTAs on expression of Bax in TNF-*α*-induced MH7A cells. MH7A cells were exposed to 20 ng/ml TNF-*α* and intervened by corresponding concentrations of FTAs for 24 h, and then the immunofluorescence of Bax was observed by Nikon fluorescence microscope system. The experimental results were recorded as mean ± SD (*n* = 3). ^#^ *p* < 0.05, ^##^ *p* < 0.01 vs. control.

In order to further explore the potential targets involved in the promoting effect of FTAs on apoptosis, western blot and immunofluorescence were employed to analyze the levels of apoptosis-associated proteins in mitochondrial apoptosis signaling pathway. As demonstrated in Figure 7, compared with TNF-*α* single treatment group, FTAs significantly increased the protein signal intensity of Bax (except for 1 μg/ml) and caspase-3, while Bcl-2 (except for 1, 10 μg/ml) was decreased on the contrary. As demonstrated in Figure 8, further observation by immunofluorescence showed that various concentrations of FTAs could increase the level of pro-apoptotic marker including Bax.

### Fuzi total alkaloids hindered excessive cell proliferation by modulating cell cycle progression

**FIGURE 9:**
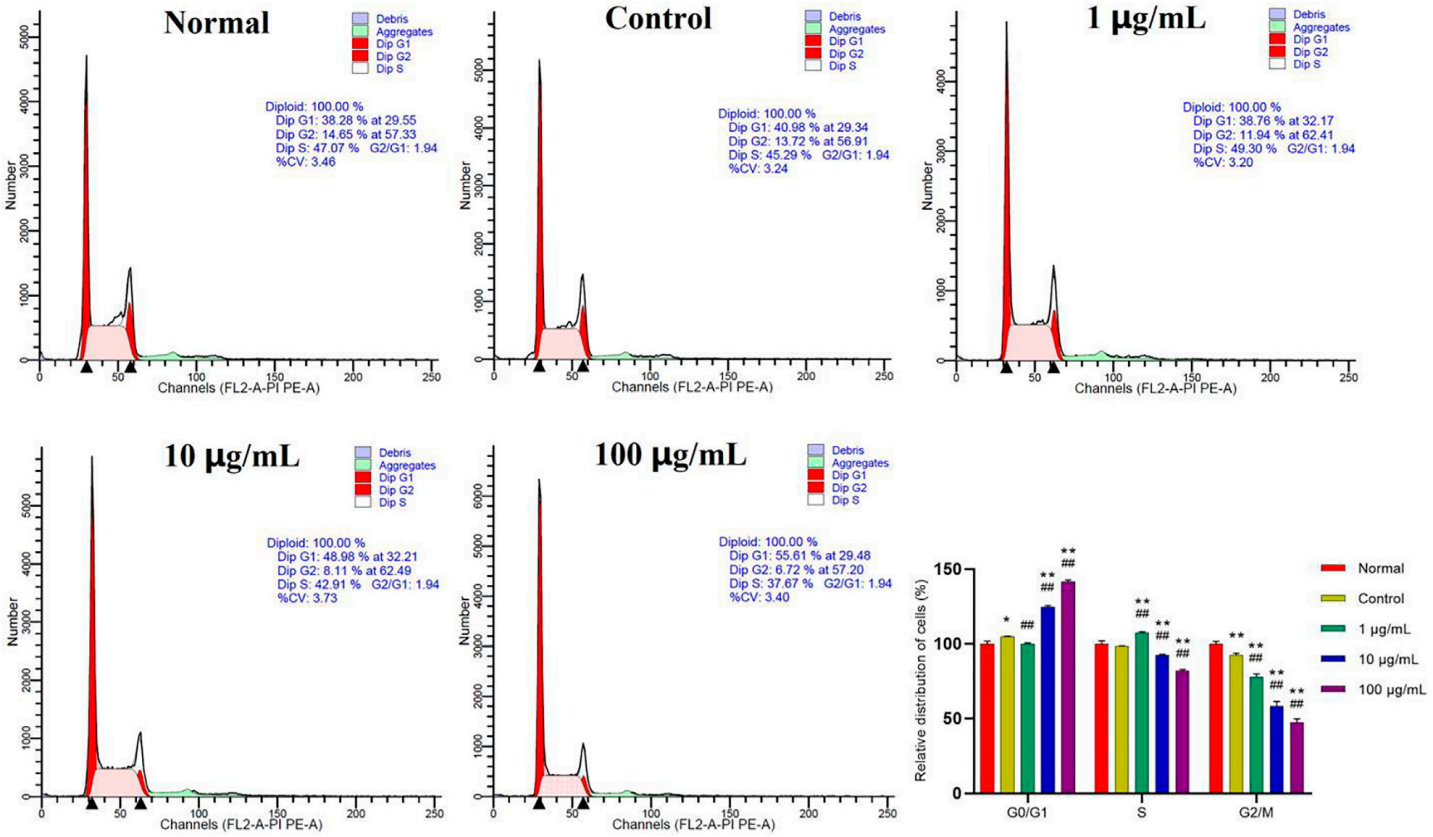
The effects of FTAs on cell cycle of TNF-*α*-induced MH7A cells. MH7A cells were intervened by corresponding concentrations of FTAs in absence or presence of 20 ng/ml TNF-*α* for 24 h, and then the cell cycle of MH7A cells was measured by flow cytometer. The experimental results were recorded as mean ± SD (*n* = 3). *p < 0.05, **p < 0.01 vs. normal, ^#^ *p* < 0.05, ^##^ *p* < 0.01 vs. control.

In addition to focusing on the effects of FTAs on apoptosis, flow cytometry was also used to investigate whether FTAs interfered with the cell cycle of MH7A cells. As demonstrated in Figure 9, compared with normal and TNF-*α* single treatment groups, different concentrations of FTAs could significantly increase the number of cells arrested at G0/G1 phase (except for 1 μg/ml), while decrease the number of cells arrested at S (except for 1 μg/ml) and G2/M phases, respectively. The above results indicated that FTAs could exert anti-RA effects by arresting the G1/S transition, and thus inhibited the hyperproliferation of MH7A cells.

## Discussion

Chinese herbal medicine has the pharmacological characteristics of “multiple components, multiple targets, multiple links and multiple effects,” and has irreplaceable capability in the treatment of various difficult and miscellaneous diseases (Lu et al., 2021). Under the guidance of compatibility theory of TCM, Fuzi appears in many anti-RA prescriptions. Among which, Fuzi plays the role of “Monarch drug,” and is the main contributor in the treatment of RA. While other herbs act as “Minister drug,” and can enhance the curative effects of Fuzi (Luan et al., 2020). Our previous study demonstrated that the alkaloids extract from Fuzi had strong anti-inflammatory activity, which significantly alleviated disease progression and reversed metabolic disorders in AIA model rats. In the present study, we used MH7A cells for *in vitro* experiments to further investigate the pharmacological mechanisms of Fuzi.

Cytokines are important mediators in regulating immune response, immune pathology and cell differentiation. In the pathophysiological process of RA, the imbalance of cytokines may cause immune cells to infiltrate synovium and joint tissues (Clavel et al., 2008). The activation of multiple signaling pathways can trigger abnormal synthesis and release of cytokines. In addition, immune complexes formed by RF and anti-CCP antibody induce macrophage activation, which is another important factor in abnormal expression of cytokines. These cytokine-mediated pathways are the core mechanism of RA pathogenesis and are considered as potential therapeutic targets (Bui and Brahn, 2019). Multiple cytokines such as IL-1*β*, TNF-*α*, IL-6, IL-17, and IL-23 are closely related to the bone metabolism, inflammation and immune processes of RA. For example, IL-1*β* induces inflammation by activating monocytes and macrophages. It can also induce the fibroblasts proliferation, osteoclasts and chondrocytes activation, resulting in synovial hyperplasia, bone absorption and cartilage damage (Kondo et al., 2021). TNF-*α* and IL-6 are involved in the induction of osteoclastogenesis. Due to abnormal expression of the above cytokines in RA patients, osteoclasts are dysfunctional in regulating bone mineral density and bone absorption, ultimately leading to bone erosion (Okamoto and Takayanagi, 2019). After being activated by pro-inflammatory cytokines, synovial fibroblasts can further secrete a variety of pathogenic molecules, such as MMPs and PGE2, and promote the transformation of early inflammation to arthritis (Schinocca et al., 2021). The abnormal increase of MMPs family members including MMP-1 and MMP-3, exerts a leading role in the degradation of articular matrix, and causes the permanent damage of bone, cartilage and tendon tissues (Zhang et al., 2020). While PGE2 acts as a mediator of inflammation and pain in RA (Wang et al., 2020). In addition, angiogenesis is related to joint inflammation in RA, and IL-6 can further increase the expression of VEGF, which induces angiogenesis in the early stage of RA progression (Guo et al., 2018). TGF-*β* exerts a complex immunomodulatory effect in the progression of RA disease, and numerous evidences indicate that TGF-*β* is capable of inducing cell proliferation, differentiation, migration, apoptosis and even angiogenesis, bone and cartilage repair and destruction. However, the relationship between it and the pathophysiological process of RA needs further study (Gonzalo-Gil and Galindo-Izquierdo, 2014). The above experimental results confirmed that FTAs could significantly reduce the expression levels of IL-1*β*, IL-6, MMP-1, MMP-3, PGE2, TGF-*β,* and VEGF in TNF-*α*-induced MH7A cells, suggesting that FTAs exert anti-inflammatory functions by regulating the synthesis and release of these cytokines.

The activation of NF-κB transcription family members is one of the key links to induce chronic inflammatory changes in RA. The NF-κB signaling pathway is activated in synovial tissues of RA patients and *in vitro* cultured synovial fibroblasts. Studies in animal models of arthritis also suggest that NF-κB exhibits a strong driving force in the pathophysiological process of RA (van Loo and Beyaert, 2011). Clinical studies have shown that the manipulation of NF-κB signaling pathway significantly alleviates inflammatory responses and arthritic symptoms in RA patients (An et al., 2020). Some intracellular factors closely related to RA process, such as IL-1*β*, IL-6, TNF-*α,* and MMPs, can directly or indirectly induce related receptor proteins to activate NF-κB (Li et al., 2014). During its activation, NF-κB can further induce the expression of RA-related proteins and genes, promote the differentiation of immune cells and regulate the intracellular inflammatory factors (Li et al., 2020). In addition, NF-κB also regulates the apoptosis signaling pathway and affects the apoptosis of fibrous synovial cells. For example, Ang II and AT1 activate NF-κB, resulting in decreased expression of caspase-3, while inhibition of NF-κB increases the expression of pro-apoptotic proteins (Pattacini et al., 2007).

Similarly, JAK/STAT signaling pathway has a non-negligible ability in chronic inflammation, which is also a potential therapeutic target for RA, and biological agents such as JAK inhibitors have been put into clinical application (Taylor et al., 2017). Different JAK subtypes and downstream STAT proteins are expressed in synovial cells and synovial tissues (Migita et al., 2013). In the non-disease state, JAK/STAT signaling reflects the effects of negative regulators of JAK/STAT, such as STAT-activating protein inhibitors and cytokines signaling inhibitors. However, RA progression is accompanied by dysfunction of these regulatory factors, leading to continuous activation of JAK/STAT signaling in inflamed synovial tissues, then enhancing the gene expression of MMPs, and increasing the degree of chondrocytes apoptosis. The most representative is the occurrence of “apoptotic resistance” in synovial cells (Malemud, 2018). The activated JAK/STAT signaling pathway promotes the signal transduction of various cytokines and molecules, which is an important driving force of autoimmune diseases such as RA. Specific cytokines dependent on the JAK/STAT pathway can induce the expression of IL-1, IL-6, TNF-*α,* and IL-17, which are closely associated with RA. Among them, TNF-*α* and IL-17 synergistically increase the synthesis and release of MMPs, thereby activating chondrocytes and FLSs (Ciobanu et al., 2020). The above experimental results confirmed that FTAs significantly suppressed the phosphorylation process of NF-κB signaling pathway and the nuclear translocation of p-p65, they also had significant regulatory effects on the activation of JAK/STAT signaling pathway. However, when IKKs and STAT3 were blocked by TPCA-1, the inhibitory effects of FTAs on IL-1*β* and IL-6 were still observed, but the efficacy was significantly reduced. Similar to other Chinese herbal medicines, FTAs have “multi-components” and “multi-targets” characteristics and are involved in regulating many signaling pathways, but according to the above results, it is certain that NF-κB and JAK/STAT are the important regulatory pathways of FTAs that exert anti-RA effects.

In healthy humans, a delicately regulated process of apoptosis is essential for coordinating the growth and death of tissue cells within the body. However, in RA patients, FLSs have apoptotic resistance, leading to synovial hyperplasia accompanied by the release of related cytokines (Zhao et al., 2021). The proliferation of synovial lining layer is often termed “pannus,” which is caused by the abnormal expansion of macrophage-like and synovial fibroblasts-like cells. The hyperplastic synovial tissues erodes the periarticular bone at the bone-cartilage boundary, leading to invasion, destruction and degradation of bone and cartilage (Aletaha and Smolen, 2018). Bcl-2 and Bax are two critical regulatory sites within mitochondrial apoptosis pathway. In the mitochondria, Bcl-2 suppress the release of cytochrome C and the activation of caspase, while Bax induce polymeric pores formation to facilitate the release of cytochrome C and activate the cascade reaction of caspase, leading to the process of cell membrane abscistion, nuclear fragmentation, and even death (Zhai et al., 2018). Activated caspase-3 is a key executor of cell apoptosis, and PARP is mainly cleaved by caspase-3. Inactivated PARP will accelerate cell instability and further induce cell apoptosis (Yan et al., 2015). Current studies indicate that apoptosis is a potential therapeutic target for RA, and inducing apoptosis of FLSs is an important molecular mechanism of Chinese herbal medicine in the treatment of RA (Zhang et al., 2019; Zheng et al., 2020). In this study, CCK-8 assay showed that FTAs inhibited proliferation of TNF-*α*-induced MH7A cells, and the inhibitory effects enhanced with higher dose. Meanwhile, FTAs promoted apoptosis of MH7A cells by acting on mitochondrial apoptosis signaling pathway, and guided cell cycle arrest at G0/G1 phase to prevent excessive cell proliferation. The results suggested that FTAs could exert anti-RA effects by inducing cell apoptosis and suppressing cell proliferation.

## Conclusion

**FIGURE 10:**
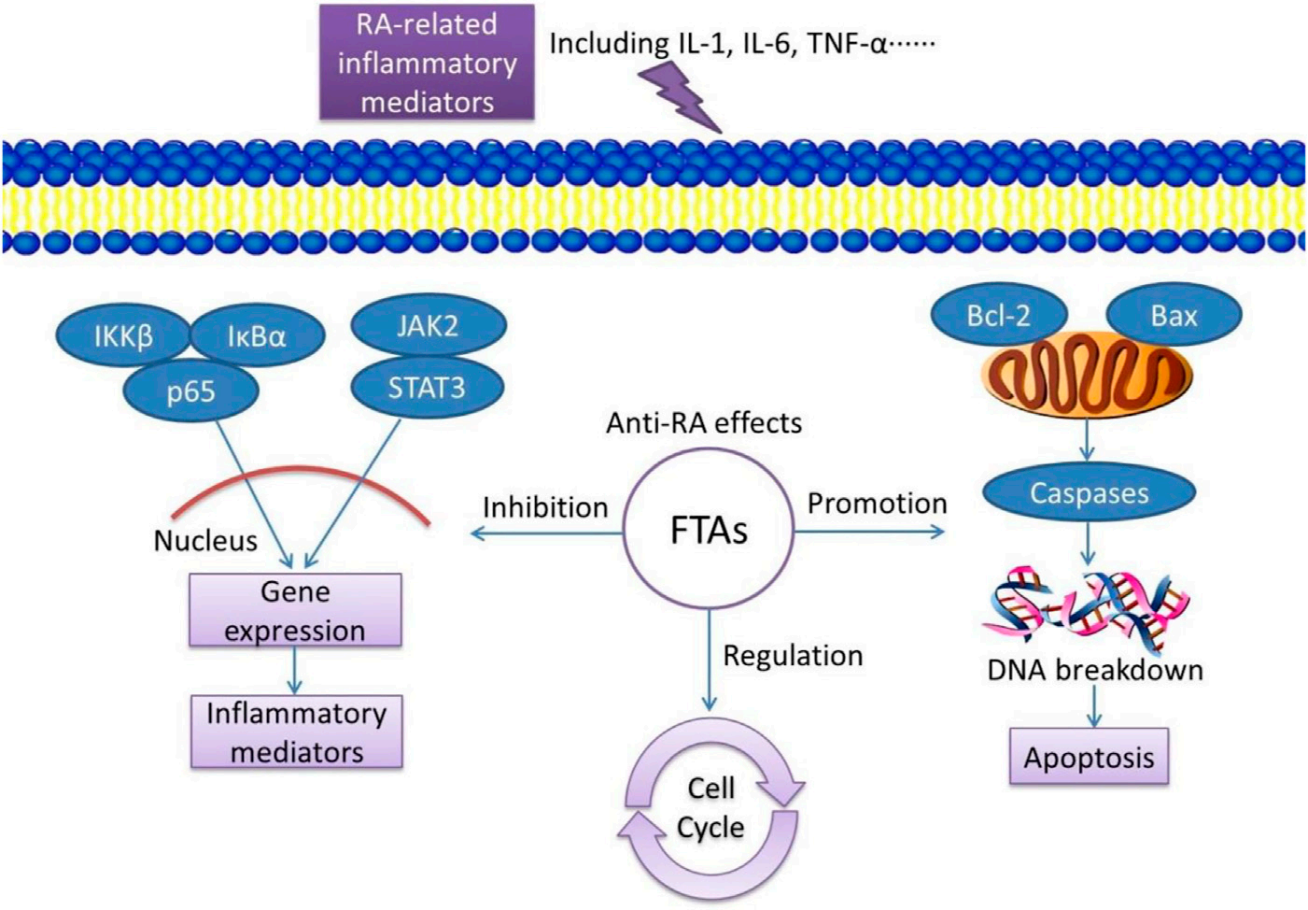
Schematic diagram of FTAs exert anti-RA effects by regulating inflammatory signaling pathways, promoting cell apoptosis and inhibiting cell proliferation.

To sum up, these experimental results confirmed that FTAs could treat RA by reversing the imbalance of pro-inflammatory cytokines, preventing excessive cell proliferation and promoting apoptosis. While, NF-κB, JAK/STAT and mitochondrial apoptosis signaling pathways were the main regulatory targets (Figure 10). This study interpreted the rationality of Fuzi against RA under the guidance of TCM theory by revealing the potential molecular mechanisms. These findings are of great significance to the modernization of Chinese herbal medicine in the field of rheumatism.

## Data Availability

The original contributions presented in the study are included in the article/supplementary material, further inquiries can be directed to the corresponding author.

## Abbreviations

FTAs, Fuzi total alkaloids; IL-1β, interleukin-1beta; JAK/STAT, Janus kinase/signal transducer and activator of transcription; MMPs, matrix metalloproteinases; NF-κB, nuclear factor kappa-B; RA, rheumatoid arthritis; PARP, poly (ADP-ribose) polymerase; PGE2, prostaglandin E2; TCM, traditional Chinese medicine; TGF-β, transforming growth factor-beta; TNF-α, tumor necrosis factor-alpha; TPCA-1, (2-[(aminocarbonyl)amino]-5-(4-fluorophenyl)-3-thiophenecarboxamide); UPLC-MS, ultra performance liquid chromatography tandem mass spectrometry; VEGF, vascular endothelial growth factor.
